# A COMMUNITY HEALTH EDUCATION PROGRAM TO ENCOURAGE EXERCISE AND PROPER NUTRITION IN LATINOS

**DOI:** 10.1093/geroni/igz038.522

**Published:** 2019-11-08

**Authors:** Karen Kopera-Frye, Ashley Graboski-Bauer, Ana Luisa Gonzalez-Marquez

**Affiliations:** New Mexico State University, Las Cruces, New Mexico, United States

## Abstract

New Mexico now has the 32nd highest adult obesity rate (28.8% for 2015). Thirty one percent of this obesity rate encompasses Latinos; rates are the highest among the 26-44 years age group. Obesity rates in NM for children aged 2-11 range from 11 – 14%. Healthy Kids New Mexico adopted the 5-2-1-0 challenge. (American Academy of Pediatrics) which refers to eating 5+ vegetables, reducing screen time to 2 hours, 1 hour+ daily activity, and daily water intake (H20). Therefore, project I’M HIP2’s main goal was to educate parents and children, within an intergenerational context, on improving diet and activity levels to reduce obesity. The Intergenerational Mentoring on Health Information Pathways 2 (I’M HIP2) program involved 74 Hispanic families including one target child, one parent, and one other relative, e.g., aunt or grandparent. Monthly educational sessions focused on physical activity and adapting meals to be healthy. Project outcomes included exercise frequency, Body Mass Index (BMI), and a knowledge quiz assessing healthy meal facts, exercise knowledge via a 10-item quiz; all assessments pre- and post-program. Paired t-test analyses revealed significant changes in knowledge quiz total scores (t 70 = 5.03, p < .0001), increased exercise frequency (t 72 = 2.106, p < .05); no significant change in BMI from pre- to post-assessments. The families reported overwhelmingly positive responses to how the program had changed their eating styles and activity levels. This study has implications for how we can harness this invaluable resource of the generations to affect health impacts on Hispanic families.

